# Mechanisms underlying the antidepressant response and treatment resistance

**DOI:** 10.3389/fnbeh.2014.00208

**Published:** 2014-06-27

**Authors:** Marjorie R. Levinstein, Benjamin A. Samuels

**Affiliations:** Department of Psychiatry, New York State Psychiatric Institute, Columbia University Medical Center, Research Foundation for Mental Hygiene, Inc.New York, NY, USA

**Keywords:** mood, depression, mouse models, treatment resistance, antidepressants, SSRI

## Abstract

Depression is a complex and heterogeneous disorder affecting millions of Americans. There are several different medications and other treatments that are available and effective for many patients with depression. However, a substantial percentage of patients fail to achieve remission with these currently available interventions, and relapse rates are high. Therefore, it is necessary to determine both the mechanisms underlying the antidepressant response and the differences between responders and non-responders to treatment. Delineation of these mechanisms largely relies on experiments that utilize animal models. Therefore, this review provides an overview of the various mouse models that are currently used to assess the antidepressant response, such as chronic mild stress, social defeat, and chronic corticosterone. We discuss how these mouse models can be used to advance our understanding of the differences between responders and non-responders to antidepressant treatment. We also provide an overview of experimental treatment modalities that are used for treatment-resistant depression, such as deep brain stimulation and ketamine administration. We will then review the various genetic polymorphisms and transgenic mice that display resistance to antidepressant treatment. Finally, we synthesize the published data to describe a potential neural circuit underlying the antidepressant response and treatment resistance.

## Introduction

Understanding the neurobiological basis of a highly complex disease like depression remains one of the foremost challenges for modern psychiatry. In patients, the essential feature of a major depressive episode is a period of at least 2 weeks during which there is either depressed mood or the loss of interest or pleasure in nearly all activities (American Psychiatric Association, 2013). These episodes are recurrent in the majority of cases. The twelve-month prevalence of major depressive disorder is approximately 7% in the US and approximately 32–35 million adults in the US population (16%) experience an episode of major depression in their lifetime (Kessler et al., 2003). Depression is ultimately associated with shorter life spans and greater risk of heart disease (Kessler, 2009). However, symptoms are diverse and vary from patient to patient. In addition to psychotherapy, several approved classes of drugs with antidepressant activity have been developed, including selective serotonin reuptake inhibitors (SSRIs), tricyclic antidepressants (TCAs), norepinephrine reuptake inhibitors (NRIs), and monoamine oxidase inhibitors (MAOIs) (Samuels et al., 2011). Furthermore, various other interventions have demonstrated efficacy for depression, including electroconvulsive therapy (ECT), vagus nerve stimulation (VNS), and transcranial magnetic stimulation (TMS) (Holtzheimer and Mayberg, 2011a).

Unfortunately there are two major problems with these interventions. First, for the antidepressants, there is a significant delay between the start of treatment and any beneficial effects (Samuels et al., 2011). SSRIs, which are the most commonly prescribed class of antidepressant, do not show beneficial effects until 2 weeks after the start of treatment and these effects are not maximal until 6–9 weeks after the start of treatment (Artigas et al., 1996; Gardier et al., 1996). Thus, one major goal of current research is to develop faster acting antidepressants. Although this work is in its infancy, potential faster acting antidepressants include Serotonin Receptor 4 (5-HT4) agonists and ketamine (Berman et al., 2000; Lucas et al., 2007; Mendez-David et al., 2013a). The second major issue with current interventions is treatment-resistance. Response rates for commonly used antidepressants are low, and remittance rates are even lower. There are many potential reasons for these low rates, including the fact that diverse sets of symptoms can all result in a depression diagnosis and, as discussed later, the genetics underlying depression are extremely complex. The largest study on response and remittance to antidepressants was a 7-year program known as sequenced treatment alternatives to relieve depression (STAR^*^D). In the STAR^*^D study, unipolar depression patients were enrolled in a multistep treatment program. All steps lasted for 12 weeks or until the patient could not tolerate the treatment. The first step for all patients was treatment with citalopram, a common SSRI. Only 28% of patients demonstrated remission with citalopram treatment as determined by the Hamilton Rating Scale of Depression (HRSD_17_), and only 33% demonstrated remission as determined by the Quick Inventory of Depressive Symptomatology-Self-Report (QIDS-SR_16_) scale (Rush et al., 2006a; Warden et al., 2007). These response rates to citalopram were similar to previous studies (Trivedi et al., 2006a,b; Warden et al., 2007). If the patients did not remit and wanted to continue in the study, they were then randomly assigned a treatment course in step 2. These treatment courses included either adjunctive therapies with either the atypical antidepressant bupropion, the Serotonin 1A receptor partial agonist buspirone, or cognitive therapy, or medication switches to bupropion, the SSRI sertraline, the NRI venlafaxine, or cognitive therapy. Only about 25% of participants entering step 2 remitted according to QIDS-SR_16_ (Rush et al., 2006b; Trivedi et al., 2006a; Warden et al., 2007). The different treatment courses all resulted in similar remittance profiles. Remittance rates were even lower for steps 3 (patients were either switched to the second generation TCA nortriptyline or the noradrenergic and specific serotonergic antidepressant (NaSSA) mirtazapine or were augmented with lithium, nortriptyline, sertraline, or venlafaxine) and 4 (patients were switched to the MAOI tranylcypromine or mirtazapine combined with venlafaxine). Similar to step 2, the different treatment courses all resulted in similar remittance profiles for steps 3 and 4. In addition, the patients that took more steps to remit or respond displayed more relapses in a long-term follow-up study (Rush et al., 2006a). Overall, approximately 20% of patients remain symptomatic despite multiple, and often aggressive, interventions (Rush et al., 2006a; Holtzheimer and Mayberg, 2011a,b). Thus, another major goal of current research is to find targets for better antidepressant treatments that will achieve and maintain remission.

Patients that do not show any response to any of the steps of treatment suffer from treatment-resistant depression (TRD). TRD is generally defined as depression that does not respond to two or more antidepressants from different classes given adequate time, dose, and compliance. However, these classes of antidepressants are usually limited to drugs that target monoaminergic neurotransmitters. More effective medications or adjunctive therapies will rely on basic research to identify targets that are distinct from monoaminergic signaling. To begin to understand TRD at a basic research level, first it is essential to develop animal models with relevant phenotypic features to reveal treatment responsiveness (Samuels et al., 2011). Importantly, it is very difficult to use rodent models to study resistance to multiple different treatments. However, rodent models are advantageous to study non-responsiveness to an initial line of treatment because molecular parameters such as protein and gene expression can be directly compared across multiple brain regions between responders and non-responders.

## Animal models of depression and treatment resistance

No genetic variants with high penetrance that cause depression are known (Lohoff, 2010; Samuels et al., 2011). Thus, several animal models of depression were developed based on whether the animals showed a response to antidepressants. While this approach has its uses, it is incompatible with attempts to study TRD. In the last decade there has been a move toward using animal models that rely on different means of chronically exposing rodents to stressful experiences. These manipulations induce states that present depression- and anxiety-like characteristics in a wide variety of behavioral tests. Subsequently, the animals can then be treated with antidepressants to test for responsiveness. Thus, these models achieve considerable face and construct validity and do not suffer from a flawed approach of being based solely on responsiveness to antidepressants. Importantly, these manipulations result in models of both anxiety and depression. While anxiety and depression are generally conceived of as distinct psychiatric disorders, they have a high comorbidity with co-occurrence rates up to 60% in patients (Gorman, 1996; Leonardo and Hen, 2006). Ultimately, given that depression is a highly heterogenous disease, no one animal model will accurately replicate the various combinations of phenotypes that are seen in depression. Successful studies will analyze data and utilize the beneficial aspects from several different animal models.

### Chronic mild stress

Chronic mild stress (CMS) was initially developed in rats using multiple stressors as a behavioral model of anhedonia that could be reversed by treatment with a TCA (Katz, 1982). Further work demonstrated that variations using more mild stressors also yielded depression- and anxiety-like characteristics, that mice were also susceptible to chronic stress, and that the induced depression- and anxiety-like characteristics could be reversed by chronic treatment with multiple classes of antidepressants (Willner et al., 1987; Willner, 2005; Surget et al., 2008).

There are a few variations of CMS. The classic CMS involves a protocol where animals are subjected to different types of mild stressors for several weeks (Willner et al., 1987; Willner, 2005; Hill et al., 2012). Some of these stressors include cage tilting, frequent bedding changes, light-dark cycle changes, and/or the presence of predator scents. These stressors are typically given in a repeated pattern for several hours per day. However, classic CMS is no longer widely used because of difficulties replicating results across laboratories (Nestler et al., 2002). Modified protocols, including chronic unpredictable stress (CUS) and unpredictable chronic mild stress (UCMS) are more useful. In both CUS and UCMS, several stressors are presented and the pattern is changed so that animals are unable to predict the next stressor. This unpredictability increases the amount of stress that the animals experience. CUS and UCMS appear to have ecological validity as they mimic stressful life experiences that may precipitate depression in humans (Mineur et al., 2006; Hill et al., 2012).

Importantly, different mouse strains have different levels of sensitivity to the UCMS procedure. Some strains do not develop a depressive phenotype at all while others display robust depression-like behavior (see Supplementary Table 1 for an overview). Overall, BALB/c mice are the most sensitive to UCMS, while C57BL/6 mice are only slightly susceptible (Ibarguen-Vargas et al., 2008; Yalcin et al., 2008; Schweizer et al., 2009). However, even within strains there are differences in susceptibility over various behavioral and cellular readouts. Thus, care must be taken when choosing a strain for UCMS experiments.

Importantly, UCMS animal models can be used to model and assess treatment resistance. Wiborg and colleagues found that a UCMS variant significantly decreased sucrose consumption and neurogenesis in rats (Jayatissa et al., 2006). Chronic treatment with escitalopram (a SSRI) resulted in a bimodal distribution in which one group showed increased sucrose consumption while the other did not. There was a correlation between the animals that showed increased sucrose consumption and reversal of the neurogenesis deficit. A follow-up study used a proteomic approach to compare the ventral hippocampus between responders and non-responders to antidepressant treatment (Bisgaard et al., 2007). The authors found that DRP-2 (dihydropyrimidinase-related protein 2) is a potential biomarker for escitalopram resistance. In a different study, Isingrini and colleagues recently used UCMS to model resistance in mice to fluoxetine (a SSRI) (Isingrini et al., 2010). The authors divided mice into several groups: fluoxetine/regular diet, fluoxetine/high fat diet, saline/regular diet, and saline/high fat diet. Each of these groups was then subjected to either control or multiple UCMS protocols. Depression-like behaviors were tested before and after each of the UCMS periods by assessing coat state, splash test results, and behavior in a reward maze test. The UCMS procedures resulted in depression-like behaviors in both the regular and high fat diet groups. However, fluoxetine treatment only reversed the effects of UCMS in the regular diet group. This suggests that diet correlates with response to antidepressant treatment. Taken together, these studies strongly suggest that UCMS is a reasonable animal model for studying non-responders to antidepressant treatment.

### Social defeat

Social defeat is an emerging animal model of depression. While schedules may differ by experiment, the basic principles are the same. An experimental animal is placed in the home cage of a bigger and more aggressive strain for several minutes at a time. This is usually done repeatedly over a course of several days with different aggressors each time, resulting in multiple experiences of defeat from the more aggressive strain. At the end of the procedure, the experimental animal is tested for social interaction by being placed in a home cage of the aggressive strain, but without the threat of being attacked or defeated. Interestingly, in this model, some of the animals appear resilient to the depressive phenotype while others exhibit susceptibility (Krishnan et al., 2007). In addition, the phenotypes induced by social defeat in susceptible mice can be reversed with antidepressant treatment (Tsankova et al., 2006).

While treatment-resistance has not specifically been assessed in animals that are susceptible to social defeat, there are parallels between responders and non-responders to antidepressant treatment and animals that are resilient and susceptible to social defeat, respectively. It is even possible that molecular studies into the differences between resilience and susceptibility may provide biomarkers that can help to predict antidepressant response. Therefore, a review of the studies into resilience and susceptibility is provided below.

Several studies have found that there are specific gene regulation and methylation differences between mice that are susceptible and resilient to social defeat. The majority of these studies come from Nestler and colleagues and focus on differences in the nucleus accumbens (NAc), a brain region in which manipulations can exert dramatic effects on depression-related behaviors (Willner, 1983; Zacharko and Anisman, 1991; Willner et al., 1992; Nestler and Carlezon, 2006). There is a genome-wide increase in dimethylK9/K27-H3 in gene-promoter regions immediately upstream of transcription start sites in animals that displayed the susceptible phenotype (Wilkinson et al., 2009). Social defeat also increases phospho-CREB close to transcription start sites and decreases phospho-CREB that is distant from start sties in susceptible animals. Interestingly, chronic treatment with imipramine (a TCA) reverses the H3-methylation and phospho-CREB changes in susceptible animals. Resilient mice show similar H3-methylation and phospho-CREB binding as control animals. In a follow-up study, Nestler and colleagues found downregulation of dishevelled-2 (DVL) protein in the NAc of susceptible, but not resilient, mice (Wilkinson et al., 2011). Blockade of DVL increases susceptibility to social defeat. Interestingly, the mechanisms underlying resilience to social defeat have also been investigated. Induction of the transcription factor ΔFosB in the NAc is necessary and sufficient for resilience (Vialou et al., 2010). Interestingly, fluoxetine-mediated induction of ΔFosB in the NAc is also necessary for reversal of the behavioral effects of social defeat in susceptible mice (Vialou et al., 2010). Taken together, these studies begin to address the molecular mechanisms underlying susceptibility to social defeat. In addition, the ΔFosB study suggests that there are direct parallels between mechanisms underlying resilience and the response to antidepressants.

In addition to these molecular studies, there are also other studies that have assessed differences in the neural circuitry between mice that are resilient and susceptible to social defeat. *In vivo* recordings found that high levels of activity in the ventral tegmental area (VTA), a midbrain region containing dopaminergic neurons that project to the NAc and other areas, are associated with increased susceptibility to social defeat (Cao et al., 2010). Interestingly, chronic fluoxetine treatment decreases firing and bursting rates of dopaminergic neurons in the VTA of susceptible mice but not control mice. A follow-up optogenetic study further clarified that induction of phasic, but not tonic, firing in the VTA induces a susceptible phenotype to social defeat (Chaudhury et al., 2013). Furthermore, specific optogenetic inhibition of the VTA-NAc projection induces resilience, while inhibition of the VTA-medial prefrontal cortex (mPFC) projection induces susceptibility. Taken together, these studies suggest that susceptibility and resilience to social defeat are encoded by a circuit containing VTA projections to the NAc and mPFC, and provide a framework for identifying the neural circuitry underlying the brain's response to stress. It will be interesting to further assess if this same circuitry is involved in mediating response to antidepressants.

### Chronic corticosterone

Several labs have mimicked the effects of chronic stress in animals through administration of glucocorticoids (Ardayfio and Kim, 2006; Gourley et al., 2008a,b,c; Murray et al., 2008; David et al., 2009). Glucocorticoid hormones are secreted by the adrenal gland in response to stress (McEwen, 1999). Therefore, chronic administration of chronic corticosterone (CORT), a glucocorticoid, can model depression in animals. CORT is a stress hormone that is analogous to cortisol in humans, and serum levels of CORT are increased in stressed and depressed animals. CORT is administered either by daily intraperitoneal (IP) injections or by placement in the homecage drinking water of the animals.

Several studies have found that CORT administration results in depression- and anxiety-related behavior. Both acute and chronic CORT injections result in increased immobility in tests that are often associated with depression-related behavior, such as forced swim and tail suspension (Murray et al., 2008; Zhao et al., 2008). Chronic, but not acute, CORT treatment affects behavior in anxiety-related tasks. More specifically, chronic CORT increases emergence in the light-dark test and latency to feed in the novelty suppressed feeding (NSF) test, and decreases sucrose consumption (Ardayfio and Kim, 2006; Gourley et al., 2008c; David et al., 2009). Chronic corticosterone (35 μg/ml in the drinking water) also decreases several measures of adult hippocampal neurogenesis, a process that is necessary for the antidepressant response (Santarelli et al., 2003; Murray et al., 2008; David et al., 2009). In addition, chronic treatment with multiple classes of antidepressants, including SSRIs, TCAs, norepinephrine reuptake inhibitors (NRIs), and melatonergics, can reverse the behavioral and neurogenic effects of chronic corticosterone in most, but not all, animals (David et al., 2009; Samuels et al., 2011; Rainer et al., 2012). Taken together, these studies demonstrate that chronic corticosterone treatment provides a useful model for modeling depression in rodents with face and construct validity.

One study used the chronic corticosterone model to study a potential biomarker for predicting antidepressant treatment response. David and colleagues found that β–arrestin 1 levels in peripheral blood mononuclear cells (PBMCs) were decreased in mice exposed to chronic corticosterone. Interestingly, chronic treatment with fluoxetine reversed this decrease in β–arrestin 1 levels. Therefore, the chronic corticosterone paradigm may prove useful for screening potential biomarkers for treatment response (Mendez-David et al., 2013b).

Only one study has attempted to model TRD using corticosterone-treated mice (Samuels et al., 2014). As alluded to above, there is usually a subgroup of animals pretreated with corticosterone that do not show a response to subsequent antidepressant treatment. This subgroup is most apparent in the NSF test, which shows a bimodal distribution potentially indicative of responders and non-responders to antidepressant treatment (Samuels et al., 2011). This same subgroup of animals that has a higher latency in the NSF also shows less of a response to antidepressant treatment in the forced swim test. Therefore, this subgroup of animals, which shows a differential response across multiple behavior tests, can be used as a model of non-response to antidepressant treatment (Samuels et al., 2011, 2014). We used a microarray approach to assess differences in the dentate gyrus of these mouse models of responders and non-responders. Interestingly, when comparing the dentate gyrus of responders and non-responders, we found an overall shift in genomic tone (Samuels et al., 2014). Pathway analysis of the probe sets from this study suggest that several signaling pathways, such as TGFβ and NFκB, contain multiple genes that show significant differences in expression between responders and non-responders (Samuels et al., 2014). Further work into identifying the molecular mechanisms underlying the differences between responders and non-responders to antidepressant treatment should provide additional insight into TRD.

## Therapeutic avenues for treatment-resistant depression

As mentioned above, only 36.8% of participants in the first step of the STAR^*^D study (and only a total of 67% after several steps) achieved remission of symptoms (Rush et al., 2006a). While animal models are starting to make progress into identifying the molecular mechanisms and neural circuitry underlying TRD, new therapeutic avenues are required immediately. In addition to antidepressant drugs, several other treatments are currently used in the clinic. These include electroconvulsive therapy (ECT), transcranial magnetic stimulation (TMS), and vagal nerve stimulation (VNS) (Avery et al., 2006; Merkl et al., 2009; Bajbouj et al., 2010; Holtzheimer and Mayberg, 2011a). In recent studies, TMS elicited a 30.6% response rate and VNS elicited a 53.1% response rate and 38.9% remission rate in TRD patients (Avery et al., 2006; Bajbouj et al., 2010). Therefore, while these methods are effective for some patients, the majority of TRD patients are in need of more effective treatments. There are two procedures that are currently experimental, deep brain stimulation (DBS) and ketamine administration, that show promise as potentially better treatments for TRD.

### Deep brain stimulation

DBS involves implantation of electrodes into brain regions and subsequent stimulation. It is somewhat similar to a pacemaker in that it delivers chronic electrical pulses to regulate firing. This method was originally developed to treat Parkinson's disease, but has been expanded to other disorders such as obsessive compulsive and major depression (Mayberg et al., 2005; Perlmutter and Mink, 2006; Holtzheimer and Mayberg, 2011a). Stimulation parameters vary widely, but generally the settings are between 60–130 Hz for the frequency, 60–200 μs for the pulse width, and 2–10 volts for the amplitude (Holtzheimer and Mayberg, 2011a).

Several different brain regions have been targeted by DBS to treat TRD. In a seminal study, Mayberg and colleagues found significant antidepressant effects in four of six TRD patients when they targeted white matter tracts adjacent to the subgenual cingulate (SGC) (Brodmann area 25). The TRD patients in this study met stringent criteria for treatment resistance that was defined as failure to respond to a minimum of four different antidepressant treatments, including medications, and evidence-based psychotherapy or electroconvulsive therapy. A short-term follow-up study of 20 patients found a 60% response rate after six months of chronic DBS (Lozano et al., 2008), and a more long-term follow-up study found a 60% response rate and 50% remission after 3 years of chronic stimulation (Kennedy et al., 2011). DBS of the ventral capsule/ventral striatum (VC/VS) also produces significant antidepressant effects. A recent study found a 40% response rate after six months and a 53% response rate at the last follow-up (24 ± 15 months) in 15 TRD patients (Malone et al., 2009). NAc, which as mentioned above regulates resilience and susceptibility to social defeat, can also be targeted by DBS. An initial report found improvements in depression ratings and hedonic responses in three TRD patients that received DBS of NAc (Schlaepfer et al., 2008). Interestingly, these improvements were reversed when the NAc stimulation was turned off. A follow-up study of 10 patients found a 50% response rate after 12 months of chronic DBS of NAc (Bewernick et al., 2010). In addition to these brain regions, single case reports found antidepressant effects in TRD patients with DBS of either the inferior thalamic peduncle or the habenula (Jimenez et al., 2005; Sartorius and Henn, 2007). Taken together, these studies suggest that DBS can elicit antidepressant responses in approximately 50% of TRD patients.

While DBS is already being used in clinical experiments, animal models may still be able to help further advance this procedure. For example, if TRD is modeled in animals with chronic stress-related protocols, then techniques such as optogenetics can be used to further parse out potential sites for DBS in humans.

### Ketamine

Recent studies have found that ketamine, a NMDA receptor antagonist, elicits rapid and extended antidepressant effects. Initial studies performed in rodents found that NMDA receptor antagonists have antidepressant effects in the forced swim and tail suspension tests, in learned helplessness paradigms, and in animals exposed to chronic stress (Trullas and Skolnick, 1990; Meloni et al., 1993; Moryl et al., 1993; Papp and Moryl, 1994; Layer et al., 1995; Przegalinski et al., 1997). An initial study into the effectiveness of ketamine in four human depression patients resulted in a rapid (within 72 h) antidepressant effect relative to control infused patients (Berman et al., 2000). Thus, ketamine may be a potentially useful treatment for TRD patients.

Zarate and colleagues were the first to assess the usefulness of ketamine administration to TRD patients (Zarate et al., 2006). The subjects that received ketamine infusions (0.5 mg/kg) showed significant improvements in depression measures within 110 min of administration. The authors found a 71% response rate and 29% remission rate on the day after ketamine infusion. 35% of the patients maintained the response for at least 1 week. Given that the effects of a single dose of ketamine are transient, a follow-up study assessed the efficacy of repeated ketamine doses for TRD patients (Aan Het Rot et al., 2010). Nine out of ten participants reported a response in symptoms on the day after the initial ketamine infusion and after the sixth and final infusion. Importantly, the authors found that ketamine treatment must be maintained as eight of the nine patients that responded to ketamine relapsed 19 days on average after the final infusion (Aan Het Rot et al., 2010). Taken together, these studies show that ketamine administration is a feasible and effective treatment for patients suffering from TRD.

Based on these studies and others, ketamine seems to be equally efficacious for all depression patients, whether they had received prior medications or not. Importantly, ketamine works through a distinct mechanism (antagonism of NMDA receptors) to elicit effects than traditional antidepressants, which generally target monoaminergic neurotransmitter systems. Therefore, the effectiveness of ketamine underscores the importance of discovering distinct mechanisms for treatment of TRD. As mentioned above, TRD is generally defined as depression that does not respond to two or more antidepressants from different classes. However, since these classes of antidepressants are usually limited to traditional antidepressants that target monoaminergic neurotransmitters, drugs that target distinct mechanisms will likely be the most beneficial for TRD patients.

While ketamine looks very promising in clinical experiments, there are still obstacles to it becoming widely used for treatment of depression in clinics. Perhaps the most significant obstacle is that ketamine is an abused recreational drug that induces a dissociative anesthesia-like state (Bergman, 1999). Ketamine is a schedule III controlled substance in the United States and a schedule I narcotic in Canada. Some of the patients in the experimental trials exhibited adverse effects including perceptual disturbances and transient dissociative symptoms (Zarate et al., 2006; Aan Het Rot et al., 2010). Therefore, it is incumbent upon basic research to determine the mechanism by which ketamine elicits antidepressant actions so that safer drugs can be developed.

Since the discovery that ketamine is a fast acting antidepressant in depression patients, there have been several preclinical studies assessing its effects in rodents. An initial study found that ketamine shows rapid antidepressant-like properties in mice exposed to a learned helplessness paradigm and the forced swim test (Maeng et al., 2008). In addition, ketamine rapidly ameliorates anhedonic and anxiogenic behaviors induced by CUS in rats (Li et al., 2011). A few studies have attempted to use rodent models to assess the mechanisms underlying the antidepressant effects of ketamine. Monteggia and colleagues found that the antidepressant effects of ketamine in mice depend on eukaryotic elongation factor (eEF2) kinase-mediated rapid synthesis of brain-derived neurotrophic factor (BDNF) (Autry et al., 2011). Interestingly, eEF2 kinase inhibitors also produced rapid antidepressant-like effects in the forced swim test. In another study, Duman and colleagues reported that ketamine rapidly activates the mammalian target of rapamycin (mTOR) pathway, which in turn resulted in increased synaptogenesis in the prefrontal cortex (Li et al., 2010). Importantly, the effects of ketamine on synaptogenesis and behavior are blocked by pretreatment with rapamycin. Taken together, these studies demonstrate that ketamine also shows rapid antidepressant effects in rodents, and that eEF2 kinase and mTOR are potential targets for novel therapeutic interventions. Further preclinical studies into the mechanisms underlying the antidepressant effects of ketamine should lead to additional targets for new antidepressants that are fast acting and elicit high response rates (Krystal et al., 2013).

One hypothesis stemming from these ketamine studies and other studies is the magnesium depletion model of depression. NMDA receptors are ionotropic glutamate receptors, and at rest the ion channels are blocked by magnesium. Voltage-dependent activation is required to remove the magnesium block and permit ion flow through the NMDA receptor channel. The magnesium depletion model proposes that decreased magnesium levels result in NMDA receptor overactivity and, as a consequence, depression and anxiety-like symptoms (Zarate et al., 2013). In this model, ketamine may elicit antidepressant effects by reversing the NMDA receptor overactivity. Interestingly, a compound known as Magtein increases magnesium levels in the brain, increases synaptogenesis in the PFC, and elicits antidepressant-like effects in the forced swim test and learned helplessness paradigm (Zarate et al., 2013). Thus, drugs that increase magnesium availability in the brain may also result in rapid antidepressant effects with high response rates.

## Mechanisms underlying depression and treatment resistance

Much of what is known about the molecular mechanisms underlying depression and antidepressant treatment come from publications describing genetically mutated mice that were assessed in anxety- and depression-related behavioral tasks. There are several publications that can be sorted into categories based on the nature of the genetic manipulation. These categories include neurotrophic factors, the serotonergic system, the glutamatergic system, the dopaminergic system, the hypothalamic–pituitary–adrenal (HPA) axis, the monoamine oxidase system, the noradrenergic system, and other systems (Kreiner et al., 2013) (Supplementary Table 2). These studies have deciphered many of the mechanisms by which known antidepressants elicit their effects. Therefore, an involved discussion of all of these genetic mutants is somewhat less important in the context of understanding TRD. However, these studies can provide information about the various brain regions that should be targeted in order to have an antidepressant response and thus may be able to inform an unbiased circuit-based approach to understanding TRD. An overview of several of these published genetic mutants is therefore provided in Supplementary Table 2.

There are a few genetic mutant models that are worth discussing in more depth. These genetic models are not based on responsiveness to known antidepressants but are instead based on polymorphisms found in the human population. These include polymorphisms in the genes encoding the neurotrophic factor BDNF, the serotonin transporter (5-HTT/SERT) and the serotonin 1A (5-HT1A) receptor.

### Val66Met polymorphism in BDNF

There are many studies assessing the role of BDNF in depression-related behavior and the antidepressant response (see Supplementary Table 2 for an overview). BDNF has been such an intense focus of interest because there is a single nucleotide polymorphism (SNP) found in the human gene encoding BDNF. This SNP results in a Met substitution for Val at codon 66 (Val66Met) in the prodomain of the gene encoding BDNF. This SNP is common in humans, with an allele frequency of 20–30% in the Caucasian population (Shimizu et al., 2004). Humans that are homozygous for the Met allele have smaller hippocampal volumes and perform poorly in hippocampus-dependent memory tasks (Egan et al., 2003; Hariri et al., 2003; Szeszko et al., 2005; Bueller et al., 2006). In order to better understand the role of this SNP in depression, Francis Lee and colleagues modeled Val66Met in mice (Chen et al., 2006). Interestingly, similar to humans, mice that were homozygous for the Met allele showed an altered hippocampal anatomy. The homozygous Met allele mice also exhibited defective BDNF secretion from neurons and increased anxiety in a stressful environment. In addition, mice that were homozygous for the Met allele responded to treatment with desipramine but were resistant to treatment with fluoxetine (Chen et al., 2006). While subsequent studies in humans have provided less consistent results, a recent meta-analysis revealed a better response to fluoxetine treatment among depressed patients carrying the Val66Met heterozygous allele than patients with homozygous allele (Zou et al., 2010a,b). By contrast, it was also reported that the Val66Met mutation resulted in resistance to antidepressant treatment (Kocabas et al., 2011). Thus, resistance to treatment with certain classes of antidepressants may be mediated by the BDNF allele of the patient.

### Serotonin-related polymorphisms and mouse models

Several studies have assessed the effects of various mutations in the serotonergic system on depression and anxiety-related behavior (see Supplementary Table 2 for an overview). The most commonly prescribed class of antidepressants (SSRIs) targets SERT and ultimately results in increased levels of serotonin in the brain. Therefore, it is not surprising that multiple components of the serotonergic system are involved in mediating the antidepressant response. However, a few polymorphisms in humans that are associated with mood disorders and treatment resistance have been described for the serotonergic system.

The best understood polymorphism in the serotonergic system occurs in the promoter region of the gene encoding SERT. This polymorphism is more commonly referred to as the serotonin transporter linked polymorphic region (5-HTTLPR) (Lesch et al., 1996). Insertion or deletion of a 44-base pair (bp)-long region gives rise to short “S” and long “L” forms of the promoter region, and the “S” form is associated with lower levels of transporter expression (Lesch et al., 1996; Murphy et al., 2004). This polymorphism has been linked to vulnerability to depression when accompanied by stressful life events, increased anxiety-related measures, and resistance to antidepressant treatment (Caspi et al., 2003; Lopez-Leon et al., 2008; Munafo et al., 2009; Taylor et al., 2010). Several genetic mouse models have manipulated SERT expression, and generally mice with decreased SERT expression display more depression-like behavior, resistance to SSRIs, and altered responses to TCAs relative to wild-type mice (Holmes et al., 2002; Lira et al., 2003; Carola and Gross, 2012). Thus, whether a depressed patient carries the “S” or “L” form of SERT likely impacts whether or not they respond to treatment with traditional antidepressants.

In addition to SERT, alterations in the 5-HT1A receptor also may contribute to treatment resistance. A C(-1019)G polymorphism in the promoter region of the gene encoding the 5-HT1A receptor is associated with depression and the response to antidepressant treatment (Wu and Comings, 1999; Le François et al., 2008). Initial reports suggested that this polymorphism specifically impacted expression of the autoreceptor population present on the serotonergic neurons of the Raphe nucleus (Lemonde et al., 2003), but more recent imaging results suggest that multiple populations of 5-HT1A receptors are affected (Parsey et al., 2006). 5-HT1A autoreceptors have also been linked with the delayed effects of SSRIs. More specifically, the autoreceptors provide feedback inhibition to the serotonergic neurons that express them, and progressive desensitization of these receptors has been suggested to underlie the delay (Blier et al., 1998). While C(-1019)G polymorphism has not yet been directly recreated in rodents, Leonardo and colleagues created an inducible 5-HT1A autoreceptor knockdown line of mice (Richardson-Jones et al., 2010). These mice have normal levels of 1A autoreceptors during development but show approximately a 30% decrease in 1A autoreceptor levels upon induction in adulthood. Since this 30% decrease relative to controls is representative of the range of receptor levels seen in humans, the authors suggest that the 1A autoreceptor knockdown mice modeled a low-expressing population (1A-Low) while the control mice modeled a high-expressing population (1A-High). Raphe neurons fire at higher rates in the 1A-Low mice, and these mice also display increased physiological reactivity to stress and decreased immobility in the forced swim test relative to 1A-High mice. Interestingly, in conditions where 1A-High mice did not show a response to fluoxetine in the NSF test (even though the 5-HT1A autoreceptors were desensitized), 1A-Low mice showed a robust response. Therefore, this paper suggests that that 5-HT1A autoreceptor desensitization alone is not sufficient to induce a response and that 5-HT1A autoreceptor levels (and thus serotonin levels throughout the brain) prior to treatment may control whether or not an individual responds to treatment with traditional antidepressants.

## Defining a neural circuit underlying TRD

While some studies have been performed, overall the mechanisms underlying TRD remain unclear. In order to perform preclinical studies aimed at defining these mechanisms, first it is necessary to know where to look. One place to start is by investigating the known neural circuitry underlying mood and the response to traditional antidepressants.

### The dentate gyrus subfield of the hippocampus

While several brain regions are known to be involved in the circuitry underlying mood disorders and response to traditional antidepressants, we recently compared dentate gyrus tissue between responders and non-responders using a microarray approach. We decided to focus on the dentate gyrus for several reasons: (1) Hippocampal neurogenesis, a process occurring in the subgranular zone of the dentate gyrus, is required for the response to most antidepressants (Santarelli et al., 2003; David et al., 2009); (2) Local pharmacological manipulations, such as BDNF or Activin infusion into the dentate gyrus, yield an antidepressant-like response (Shirayama et al., 2002; Dow et al., 2005); (3) the dentate gyrus contains a relatively homogenous population of cell types relative to other brain areas.; and (4) the practical reason that we have identified a specific behavior, NSF, which requires an intact dentate gyrus and is responsive to antidepressant treatment (Santarelli et al., 2003; David et al., 2009; Samuels et al., 2014). We compared dentate gyrus gene expression between responders and non-responders and found an overall shift in genomic tone. However, we also found several specific pathways, such as TGFβ and NFκB, that may be targetable (Samuels et al., 2014).

**Figure 1:**
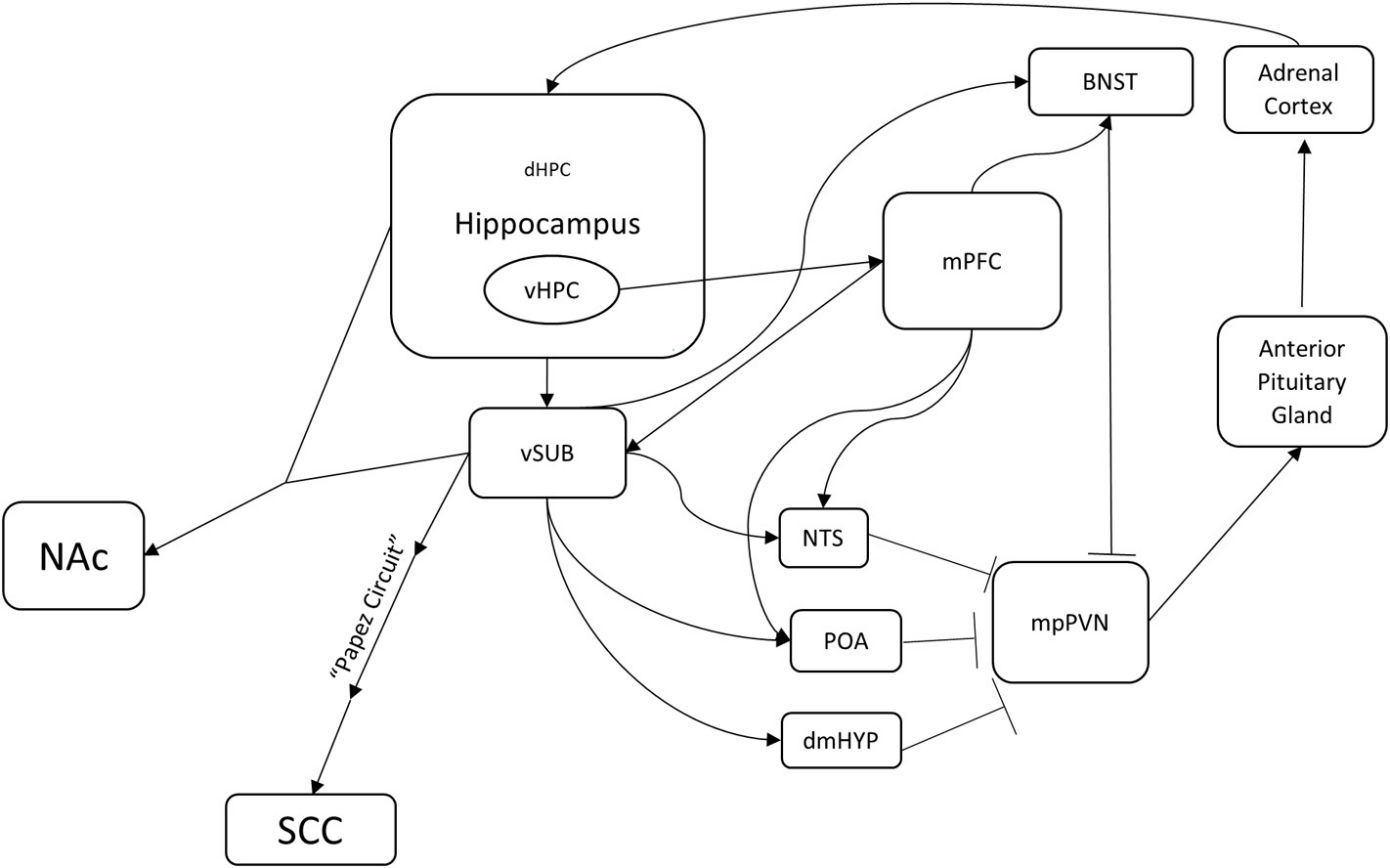
**A potential hippocampus-based neural circuit for TRD.** Several distinct neural circuits likely underlie resistance to traditional antidepressants. This is one potential hippocampus-based circuit. The hippocampus regulates the HPA axis (mpPVN > Anterior Pituitary Gland > Adrenal Cortex) through multiple brain nuclei. Medial parvocellular paraventricular nucleus (mpPVN) neurons receive inhibitory inputs from the bed nucleus of the stria terminalis (BNST), the dorsomedial hypothalamus (dmHYP), the preoptic area (POA), and the nucleus of the solitary tract (NTS). In turn, these areas receive excitatory inputs from the ventral subiculum (vSUB) and the medial prefrontal cortex (mPFC). The ventral hippocampus innervates and regulates the ventral subiculum (vSUB) and the medial prefrontal cortex (mPFC). Importantly, this is a feedback loop as corticosterone produced in the adrenal cortex regulates a large population of receptors in the dentate gyrus of the hippocampus. In addition, the ventral hippocampus (vHIP) regulates the nucleus accumbens (NAc) through the ventral subiculum (vSUB) and the subgenual cingulate (SGC) through the ventral subiculum (vSUB) and the Papez circuit. Identification of mechanisms underlying treatment resistance in this circuitry may result in novel therapeutic avenues.

One potential circuit underlying TRD may center on the dentate gyrus (and thus the hippocampus) (Figure 1). The involvement of the hippocampus in anxiety-like behavior is profound; it is central to the circuitry of the stress response. Several classic studies demonstrated that the hippocampus is involved in the regulation of mood by demonstrating the vulnerability of the hippocampus to various hormones induced by stressful experiences (McEwen, 1999). In the CA3 subfield of the hippocampus, for example, 21 days of restraint stress or corticosterone treatment leads to atrophy of apical dendrites (McEwen et al., 1995; McEwen, 1999). Adrenalectomy in adult rats causes increased death of mature granule neurons in the dentate gyrus (Sloviter et al., 1989; Gould et al., 1990). Adult dentate gyrus neurogenesis is also regulated by stress (Dranovsky and Hen, 2006; Samuels and Hen, 2011). Exposure to different forms of chronic stress, including social subordination, immobilization, physical restraint and footshock can suppress adult neurogenesis in multiple species (Gould et al., 1997, 1998; Czéh et al., 2001, 2002). In addition, optogenetic elevation of granule cell activity in the ventral dentate gyrus powerfully suppresses innate anxiety (Kheirbek et al., 2013). For all of these reasons, the hippocampus may be an entry point to defining a neural circuit underlying TRD.

### The hippocampus and mood circuitry

The most likely mechanism by which stress suppresses adult neurogenesis in the hippocampus is via activation of the HPA axis and subsequent elevation of cortisol (glucocorticoid) levels. Several lines of evidence support this hypothesis. First, adrenalectomy increases adult DG neurogenesis (Cameron and Gould, 1994). Second, both acute and chronic treatment with corticosterone leads to a decrease in neurogenesis (Gould et al., 1992; Cameron and Gould, 1994; Cameron et al., 1998; Karishma and Herbert, 2002; Murray et al., 2008). Third, glucocorticoids also inhibit the proliferation and differentiation of neural progenitors and the survival of young neurons (Wong and Herbert, 2004). Hippocampal neurons express receptors for glucocorticoids, which suggests that glucocorticoids have direct effects on the hippocampus (McEwen et al., 1968; Dranovsky and Hen, 2006). Thus, the hippocampus is strongly regulated by stress.

Interestingly, the hippocampus also provides negative feedback to the HPA axis. When the hippocampus is lesioned, basal levels of glucocorticoids increase and the stress response is prolonged (Jankord and Herman, 2008). This regulation appears to be via the major output of the hippocampus, namely the ventral subiculum (vSUB). The vSUB directly innervates GABAergic neurons in several nuclei including the bed nucleus of the stria terminalis (BNST), the nucleus of the solitary tract (NST), the dorsomedial hypothalamus (dmHYP), and the preoptic area (POA) (Herman and Mueller, 2006; Jankord and Herman, 2008; Surget et al., 2011). In turn, these nuclei directly innervate and inhibit the entry point to the HPA axis, the medial parvocellular paraventricular nucleus (mpPVN) of the hypothalamus. The mpPVN secretes corticotropin releasing hormone (CRH), which then excites the anterior lobe of the pituitary gland, which in turn releases adrenocorticotropic hormone (ACTH). ACTH activates the adrenal cortex, which results in release of glucocorticoids (Herman and Mueller, 2006). Lesions to the vSUB also enhance HPA axis responses to stress, but do not affect basal corticosterone levels (Herman and Mueller, 2006; Jankord and Herman, 2008). Therefore, the hippocampus is both a target and a regulator of the stress response.

The hippocampus also regulates regions that are known to control mood, such as the medial prefrontal cortex (mPFC), NAc, and SGC (Brodmann area 25). The ventral hippocampus (vHPC) shows theta synchrony with mPFC, which in turn regulates the stress response via activation of GABAergic neurons in BNST, NST, and POA (Jankord and Herman, 2008; Adhikari et al., 2010). Based on the timing of the theta synchrony, the signal appears to originate from the vHPC and travels to the mPFC. This synchrony increases while mice are in an anxiogenic environment such as an elevated plus maze or a novel arena (Adhikari et al., 2010). The hippocampus also innervates NAc, which as mentioned above is a region that regulates response to social defeat and is a target for DBS in TRD patients. One of the most prominent subcortical efferent projections of vSUB is to NAc, and hippocampal (CA1) and vSUB neurons demonstrate synchronous activity with medium spiny neurons in NAc (Goto and O'Donnell, 2001; Witter and Amaral, 2004). The hippocampus also regulates SGC (Brodmann area 25), a major target of DBS, through the Papez circuit, which has been studied for more than a century and is affected in many memory disorders (Triarhou, 2008). Taken together, these anatomical connections suggest that the hippocampus is a central player and regulator of the circuitry that underlies mood.

## Summary and future directions

While the mechanisms underlying resistance to antidepressant treatment remain unknown, a framework is developing for modeling and assessing treatment resistance in animals. One approach that may work is to use rodent models of depression (such as UCMS, social defeat, or CORT) to isolate responders and non-responders to antidepressant treatment. Then, the various brain regions involved in the circuitry underlying depression can be dissected and compared between responders and non-responders. We took this approach in a recent study and found several differences in the dentate gyrus (Samuels et al., 2014). Future studies using this type of approach should pave the road for novel therapeutic avenues that will supplement or perhaps even replace current antidepressant treatments.

### Conflict of interest statement

The associate editor, Dr. Kheirbek declares that, despite being affiliated with the same department as the authors, the review process was handled objectively. The authors declare that the research was conducted in the absence of any commercial or financial relationships that could be construed as a potential conflict of interest.
