# Supplementary material for: Mechanisms underlying the antidepressant response and treatment resistance
Source: Front Behav Neurosci. 2014 Jun 27;8:208. doi: 10.3389/fnbeh.2014.00208 (PMC4073308; doi:10.3389/fnbeh.2014.00208)
Supplement: Supplementary file 1 [file DataSheet1.DOCX]

***Supplementary Material***

**Mechanisms underlying the antidepressant response and treatment resistance**

Marjorie Rose Levinstein and Benjamin Adam Samuels*

Columbia University Medical Center

Department of Psychiatry

Research Foundation for Mental Hygiene, Inc.

New York State Psychiatric Institute

*** Correspondence:** Benjamin Adam Samuels, Ph.D., Columbia University Medical Center, Research Foundation for Mental Hygiene, Inc., New York State Psychiatric Institute, 1051 Riverside Dr., Box 87, New York, NY, 10032, USA

[bas2180@columbia.edu](mailto:bas2180@columbia.edu)

**Supplementary Table 1. Mouse strain susceptibility to UCMS, chronic corticosterone, and social defeat.**

| Study | Assessments | Stain | Susceptibility | Antidepressant effect |
| --- | --- | --- | --- | --- |
| *UCMS* |  |  |  |  |
| Ibarguen-Vargas et al, 2008 | Coat quality  NSF | C57BL/6 | Slightly susceptible | TCA reversed NSF deficits |
|  |  | BALB/c | Highly susceptible | TCA reversed coat, NSF deficits |
|  |  | DBA | Highly susceptible | TCA reversed coat deficits |
|  |  | A/J | Moderately susceptible | TCA reversed NSF deficits |
|  |  | C3H | Moderately susceptible | TCA Ineffective |
|  |  | CBA | Moderately susceptible | TCA Ineffective |
|  |  | FVB | Moderately susceptible | TCA Ineffective |
| Schweizer, Henniger, & Sillaver, 2009 | SPT  D/L  FST  Modified Hole board | C57BL/6J | Slightly susceptible | Untested |
|  |  | C57BL/6N | Not susceptible | Untested |
|  |  | DBA | Highly susceptible | Untested |
| Yalcin et al, 2008 | Coat quality  Splash test | Swiss Webster  BALB/c | Not susceptible  Highly susceptible | Did not significantly change behavior  Partially restored with TCA, TeCA, SSRI |
| *Chronic Corticosterone* |  |  |  |  |
| Zhao et al, 2008 | FST  TST | C57BL/6N | Susceptible at 3 and 5 weeks of administration | Untested |
| Murray et al, 2008 | FST  D/L | CD1 | Susceptible | Untested |
| Gourley et al, 2008 | SPT  Instrumental conditioning  FST | C57BL/6 | Susceptible after weaned off CORT | BDNF microinfusions reversed behavioral deficits |
| David et al., 2009 | OF  NSF  FST  Coat quality  Splash test  EPM  TST | C57BL/6Ntac  CD1 | Highly susceptible  Moderately susceptible | Fluoxetine, imipramine, and reboxetine reversed deficits in most behavioral tests |
| *Social Defeat* |  |  |  |  |
| Razzoli et al, 2011 | Social avoidance  FST  Metabolic processes  Hormone levels | C57BL/6J  BALB/c | Moderately susceptible to repeated, not single  Highly susceptible | Restored with SSRI  Restored with SSRI |
| Keeney, Hogg, & Marsden, 2001 | Corticosterone levels  Body temperature | NMRI | Moderately susceptible | Restored with SSRI |

BDNF – brain-derived neurotrophic factor; CORT – corticosterone; D/L – dark/light emergence test; EPM – elevated plus maze; FST – forced swim test; NSF – novelty suppressed feeding; OF – open field; SPT – saccharine/sucrose preference test; SSRI – selective serotonin reuptake inhibitor; TCA – tricyclic antidepressant; TeCA – tetracyclic antidepressant; TST – tail suspension test; UCMS – unpredictable chronic mild stress

**Supplementary Table 2. Genetic mutations associated with depression and treatment-resistance.**

| Study | Gene | Variation | Subjects | Behavior Tests | Result | Antidepressant Effect |
| --- | --- | --- | --- | --- | --- | --- |
| *Neurotrophic Factors* |  |  |  |  |  |  |
| Duncan et al., 2009 | BDNF | Val/Val | Human | Diagnostic interviews, self-report questionnaires | Increased severity of depression symptoms. |  |
| Boulle et al., 2012 | BDNF | Het | Mice | Social interaction after chronic social defeat. | Impaired response to antidepressants |  |
|  | BDNF | N/A | Rats | Chronic social defeat | Social defeat decreases BDNF expression and increases H3K27 methylation. |  |
| Fanous et al., 2010 | BDNF | N/A | Rats | Repeated social defeat stress | Short-term increase of BDNF protein levels in PFC after stress. Short-term increase of BDNF mRNA in amygdalar regions after stress. Elevated mRNA and protein levels in VTA 28 days after stress. |  |
| Ibarguen-Vargas et al., 2009 | BDNF | Het | Mice | Actimeter, R/I, NSF, TST, coat condition | No baseline behavior differences. | Slight decrease in antidepressant response. |
| Uchida et al., 2011 | GDNF | N/A | Mice | FST, SPT, social interaction, NSF | Decreased expression in striatum of stressed animals. However increased expression in resilient animals. |  |
| MacQueen et al., 2001 | BDNF | Het | Mice | Novel object, EPM, staircase, SPT, FST, LH, passive avoidance, hot plate, tail-flick, formalin injection | No changes in locomotion, anxiety-like behaviors or anhedonia in the het mice compared to WT. No changes in behavioral despair, but increased latency and more failed escapes in learned helplessness test in hets; however, this could be explained by decreased nociception. |  |
| Adachi et al., 2008 | BDNF | KO (HPC) | Mice | EPM, fear conditioning, SPT, FST | No differences in anxiety related behavior, fear conditioning, anhedonia, or behavioral despair. | Desipramine decreased immobility in BDNF CA1 KO. |
| Zörner et al., 2003 | trkB | CamKII Cre | Mice | OF, EOM, D/L, novel object, FST | No changes in anxiety-like behavior. Cre mice had greater latency to float and less immobility than controls in FST. |  |
| Monteggia et al., 2007 | BDNF | CamKII Cre (Forebrain) | Mice | EPM, OF, FST, SPT | No difference in anxiety-like behaviors in males, but female conditional KO spent more time in open arms than littermate controls. No difference in anhedonia or behavioral despair in males, but female conditional KO showed greater anhedonia and increased immobility in FST. | Desipramine did not decrease immobility in conditional KO. |
|  | BDNF | GFAP Cre (Forebrain) |  |  | No differences in EPM. Cre+ females displayed increased immobility in FST. |  |
| Newton et al., 2002 | CREB | OE | Mice | LH | Increase in escape failures relative to controls. |  |
|  | mCREB | OE | Mice |  | Decrease in escape failures relative to controls. |  |
| *Serotonergic System* |  |  |  |  |  |  |
| Antilla et al., 2009 | TPH2 | Polymorphisms | Human | Montgomery and Åsberg Depression Rating Scale |  | Treatment resistance to ECT. |
| Taylor et al., 2010 | SERT | Short vs. Long | Human | Meta-analysis of studies |  | Long carriers have greater response to antidepressants targeting SERT. |
| Lira et al., 2003 | SERT | KO | Mice | OF, EPM, NSF, FST, TST, Shock avoidance, SIH, foot shock sensitivity | Increased depression-like behaviors |  |
| Holmes et al., 2002 | SERT | KO | Mice | TST, FST | KO on C57BL/6J background showed no baseline differences with WTs. KO on 129S6 background showed decreased immobility in TST, but increased immobility in FST. | Fluoxetine had no effect on KO on C57BL/6J background. Desipramine decreased immobility time in TST more in KO than in WT. Imipramine decreased immobility in all genotypes. |
| Alexandre et al., 2006 | SERT | KO | Mice | TST, locomotor activity | KO displayed increased immobility in TST | WAY 100635 decreased immobility in KO. |
| Ferrés-Coy et al., 2013 | 5HT_1A_R | Selective knockdown or knockout | Mice | EPM, TST, FST | Increased anxiety-like behaviors in knockout mice. Knockout and knockdown showed decreased depression-like behaviors. Knockdown had greater serotonin expression after stress than wildtype. Fluoxetine more greatly increased serotonin levels in mPFC in knockout and knockdown. |  |
| Richardson-Jones et al, 2010 | 5HT_1A_R | Inducible autoreceptor knockdown | Mice | OF, D/L, FST, TST, NSF | After stress, induced knockdown mice showed greater depression-like behaviors than non-induced mice. | Fluoxetine treatment decreased latency in NSF even at subchronic doses. Induced knockdown had greater serotonin levels at 8 days of fluoxetine treatment, but did not differ from non-induced mice at 26 days. |
| Mayorga et al., 2001 | 5HT_1A_R | KO | Mice | TST | KO mice less baseline immobility. | SSRIs did not affect immobility. TCA reduced immobility |
| Ramboz et al., 1998 | 5HT_1A_R | KO | Mice | OF, EPM, FST | Decreased time spent in open arms. Decreased immobility. |  |
| Jones & Lucki, 2005 | 5HT_1A_R | KO | Mice | TST | KO mice showed less baseline immobility. |  |
| Heisler et al., 1998 | 5HT_1A_R | KO, Het | Mice | Home cage activity, OF, EOM, rotorod, novel object, TST | KO showed decreased time in open area, increased latency to approach novel object, decreased immobility in TST than hets and WT. Het mice also showed less immobility than WT. |  |
| Mayorga et al., 2001 | 5HT_1B_R | KO | Mice | TST | No differences of immobility at baseline between KO and WT. | Lower doses of SSRIs needed to reduce immobility in KO than WT. |
| Jones & Lucki, 2005 | 5HT_1B_R | KO | Mice | FST, TST | Female KO mice showed less immobility. Male KO mice did not differ from WT. | Fluoxetine enhanced the decreased immobility in female KO. |
| Weisstaub, 2006 | 5HT_2A_R | KO | Mice | OF, FST, TST, D/L, EPM, NSF | Decreased anxiety-like behaviors across tests in KO than WT. No difference in immobility times for FST and TST. |  |
| Bhatnagar et al., 2004 | 5HT_3_R | KO | Mice | FST, OF, defensive withdrawal | Increased immobility in KO females. Decreased time in open area in KO males. |  |
| Guscott et al., 2005 | 5HT_7_R | KO | Mice | PPI, FST | Decreased immobility in KO |  |
| Sarkisyan et al., 2010 | 5HT_7_R | KO | Mice | FST, TST, locomotor activity | KO showed decreased immobility in both FST and TST. | Citalopram further decreased immobility. Desipramine had no effect on KO. |
| *Glutamatergic System* |  |  |  |  |  |  |
| Tordera et al., 2011 | VGLUT | Het | Mice | Sucrose intake, FST | Increased depression-like behaviors. Similar gene-expression to stressed animals. |  |
| Boyce-Rustay & Holmes, 2006 | NR2A | KO | Mice | EPM, D/L, OF, FST, TST | KO displayed less anxiety-like behavior by spending more time in and having more entries into open arms in EPM and the light compartment in light/dark test. KO also had less immobility in FST and TST. |  |
| Miyamoto et al., 2002 | NR2D | KO | Mice | EPM, D/L, FST | KO had greater open arm entries and entries into the light side in EPM and light/dark respectively. KO also had less immobility in FST. |  |
| Fell et al., 2011 | mGlu2 | KO | Mice | FST | KO spent less time immobile during FST than WTs. |  |
| Inta et al., 2013 | mGlu5 | KO | Mice | OF, D/L, EOM, FST | Increase anxiety-like behavior in KO in both elevated zero maze and light/dark test. Increases in latency to and decreases in entries into the open part of the zero maze and light compartment of light/dark test were exaggerated by aging. Young KO display decreased immobility time in FST; whereas, older animals have unaltered immobility time. |  |
| Cryan et al., 2003 | mGlu7 | KO | Mice | FST, TST, D/L, EPM, staircase, SIH, passive avoidance | KO displayed less immobility in both FST and TST compared to WT. KO also had more entries into and more time spent in the light compartment or open arms of the light/dark test and EPM respectively. KO also had fewer rearings in the staircase test. No differences in SIH or passive avoidance. |  |
| *Dopaminergic System* |  |  |  |  |  |  |
| Xing et al., 2013 | D_3_ | KO | Mice | OF, EPM, D/L, FST, TST, novel cage | No changes in anxiety-like behaviors between KO and WTs. Acutely stressed KO did not show an increase in immobility in TST that was seen in WT. No other difference seen in FST or TST. |  |
| Holmes et al., 2001 | D_5_ | KO | Mice | Rotarod, OF, PPI, EPM, D/L, Morris water maze, CFC, FST | No differences in coordination, startle reflex, or anxiety-like behaviors between KO and WT. Male KO showed less immobility in FST than male WT, no differences between females. |  |
| Spielewoy et al., 2000 | DAT | KO | Mice | OF, FST, social interaction, R/I | KO mice displayed hyperlocomotion compared to WT. KO mice swam the entire FST. KO had normal social behavior |  |
| *HPA axis* |  |  |  |  |  |  |
| Ridder et al., 2005 | GR | Het | Mice | OF, EOM, D/L, FST, CFC, LH | Het mice were indistinguishable from WT at baseline in all but learned helplessness. Het mice displayed higher escape latencies and an increase in escape failures. |  |
|  | GR | OE |  |  | OE mice were indistinguishable from WT at baseline in all but learned helplessness. OE mice displayed lower escape latencies and a decrease in escape failures. |  |
| Tronche et al., 1999 | GR | Nestin Cre | Mice | FST, D/L, EOM | Cre+ mice had similar immobility times as controls in FST. Cre+ mice showed less anxiety in both elevated zero maze and light/dark box. |  |
| Chmielarz et al., 2013 | GR | DBH Cre | Mice | OF, TST, D/L | Female Cre+ mice showed increased immobility in TST and increased latency to emerge in light/dark box in unstressed conditions. Cre+ males appear stress resistant as chronic restraint stress did not alter increase anxiety-like or depression-like behaviors, unlike control counterparts. |  |
| Bale & Vale, 2003 | CRF2 | KO | Mice | FST | KO had increased immobility when compared to WT. | Antalarmin (CRF1 antagonist) decreased immobility in KO. |
| van Gaalen et al., 2002 | CRF2 | OE | Mice | D/L, OF, hole board, CFC, FST | OE had reduced immobility in FST when compared to WT. OE had decreased time spent in light side of light/dark box. |  |
| Solomon et al., 2013 | GR | CamKII Cre | Mice | FST, SPT | Male Cre+ mice displayed increased immobility and decreased sucrose preference compared to controls. Cre+ females did not differ from controls. |  |
| Chen et al., 2006 | Ucn-2 | KO | Mice | FST, TST, EPM, D/L, CFC | KO females had less immobility in both FST and TST compared to WT females. KO males did not differ in these tests compared to WT. KO males and females did not differ from WT in measures of anxiety. |  |
| *Monoamine Oxidase* |  |  |  |  |  |  |
| Cases et al., 1995 | MAOA | KO | Mice | FST | KO had decreased immobility time. |  |
| Grimsby et al., 1997 | MAOB | KO | Mice | OF, EPM, FST | KO displayed no differences in locomotion or anxiety-like behavior. KO displayed decreased immobility in FST. |  |
| *Noradrenergic system* |  |  |  |  |  |  |
| Doze et al., 2009 | α_1A_-AR | KO | Mice | FST, TST, OF, EPM, D/L | KO had decreased immobility in FST and TST compared to WT. There were no differences in anxiety-like behavior between KO and WT. |  |
|  | α_1B_-AR | KO |  |  | KO had increased immobility in FST and TST compared to WT and increased locomotor activity in open field. There were no differences in anxiety-like behavior between KO and WT. |  |
| Schramm, McDonald, & Limbird, 2001 | α_2A_-AR | KO | Mice | FST, OF, D/L | KO had increased immobility in FST compared to WT. KO spent less time in light compartment in light/dark test. | Imipramine did not reverse deficits in KO, but did decrease immobility in WT. Imipramine decreased time spent in light compartment in both KO and WT. |
| Sallinen et al., 1999 | α_2C_-AR | KO | Mice | FST | KO had decreased immobility time compared to WT. |  |
|  | α_2C_-AR | OE |  |  | OE had increased immobility time compared to WT. |  |
| Haller et al., 2002 | NET | KO | Mice | FST | At baseline there was no difference between KO and WT. After social defeat, KO had significantly less immobility than WT. |  |
| Dziedzicka-Wasylewaka et al., 2006 | NET | KO | Mice | FST, TST | KO displayed less immobility in both FST and TST compared to WT. | Reboxetine, desipramine, and imipramine had no effect on KO, while lowering immobility in WT. Citalopram decreased immobility in both KO and WT. |
| *Opioid System* |  |  |  |  |  |  |
| Filliol et al., 2000 | µOR | KO | Mice | EPM, D/L, FST | KO had more open arm entries and time spent in open arms. Similar results in light/dark test. KO had increased immobility time compared to WT. |  |
|  | δOR | KO | Mice |  | KO had fewer open arm entries and time spent in open arms than WT. Similar results in light/dark test. KO had decreased immobility time compared to WT. |  |
| *Other systems* |  |  |  |  |  |  |
| Bukh et al., 2010 | Multiple | Polymorphisms | Human | Interviews, questionnaires | No correlations to depression remission rates nor interactions. |  |
| Elliot et al., 2010 | HDAC2 | N/A | Mice | Social interaction | Increase of HDAC2 in stressed animals. | HDAC inhibitor and anti-HDAC virus in NAc decreased depression-like behaviors. |
| Tanda et al., 2009 | nNOS | KO | Mice | OF, D/L, EPM, social interaction, PPI, FST, radial arm maze, Morris water maze | No difference between KO and WT in anxiety measures. KO had increased immobility in FST. Impaired working and spatial memory in KO. |  |
| Fedorova et al., 2003 | A3 | KO | Mice | OF, D/L, EPM, FST, TST, CFC | KO had greater number of open arm entries in EPM compared to WT. KO had increased immobility in TST and FST compared to WT. |  |
| Dalla et al., 2004 | Ar | KO | Mice | OF, EPM, FST | No difference in locomotion of anxiety. KO mice (all female) had greater immobility than WT. |  |
| Lu et al., 2008 | GalR2 | KO | Mice | EPM, D/L, FST, TST, OF, LH | No differences between KO and WT in EPM, light/dark, TST, FST, and OF. KO had more escape failures in LH. | Desipramine decreased immobility in both WT and KO in FST. |
| Weil et al., 2006 | MT1 | KO | Mice | PPI, FST, OF | KO had deficits in sensorimotor gating in PPI and increased immobility in FST. |  |
| Aonurm-Helm et al., 2008 | NCAM | KO | Mice | OF, rotarod, TST, SPT, taste aversion | KO had greater immobility in TST than WT. KO had decreased sucrose preference. | Citalopram did not decrease immobility in KO. Amitriptylline and FGL decreased immobility in KO. All increased sucrose preference in KO. |
| Hashimoto et al., 2009 | PACAP | KO | Mice | FST | KO had increased immobility in FST compared to WT. | Risperidone, ritanserin and PACAP infusion decreased immobility in FST. |
| Smith et al., 2009 | Relaxin-3 | KO | Mice | PPI, Y-maze, OF, rotarod, social interaction, novel object, D/L, EPM, FST | No deficits in sensorimotor gating, spatial memory, or coordination. KO females were hypoactive and had fewer social interactions. No genotype differences in anxiety measures. KO males had greater immobility on second day of FST. |  |
| Zeng et al., 2007 | TRH-R1 | KO | Mice | OF, hot plate, PPI, CFC, EPM, FST, TST | No deficits in locomotor activity, nociception, sensorimotor gating, or fear conditioning. KO spent more time in closed arms and had fewer open arm entries in EPM. KO had greater immobility in both TST and FST. |  |
| El Yacoubi et al., 2001 | A_2A_ | KO | Mice | FST, TST, OF | KO had less immobility in both FST and TST. | A_2A_ antagonists decreased immobility in WT. |
| Krishnan et al., 2008 | AC5 | KO | Mice | EPM, D/L, OF, FST, SPT, social interaction | Male KO spent more time in light side of light/dark box than WT. Male and female KO spent more time in open arms of EPM than WT. Female KO mice spent less time immobile than WT. KO had less social interaction than WT. |  |
|  | AC1/8 | KO |  |  | No difference between KO and WT in light/dark box. KO entered closed arms of EPM more than WT. Male KO mice spent less time immobile than WT KO were more anhedonic than WT. KO had more social interaction than WT. |  |
| Mombereau et al., 2004 | GABA_B(1)_ | KO | Mice | D/L, staircase, OF, FST, TST | KO had increase in anxiety-like behaviors in light/dark box and staircase. KO had decreased immobility in FST, but no difference in TST. | GABA_B_ positive modulator reduced anxiety-like behavior and decreased immobility in FST in WT. |
| Mombereau et al., 2005 | GABA_B(2)_ | KO | Mice | D/L, FST | KO had decreased transitions and spent less time in light side of light/dark box. KO had decreased immobility in FST compared to WT. |  |
| Liu et al., 2007 | GAT1 | KO, Het | Mice | FST, TST, OF, D/L, EPM | Het had less immobility in FST and TST than WT; KO had less immobility in FST and TST than Het. KO had less anxiety-like behavior in light/dark, emergence, OF, and EPM. | Amitriptyline, imipramine, and fluoxetine decreased immobility in WT and Het in TST. Only imipramine decreased immobility in KO. |
| Rupniak et al., 2001 | NK1 | KO | Mice | R/I, FST, TST, EPM | KO were less aggressive, had less immobility in both FST and TST, and spent more time in open arms of EPM than WT. |  |
| Tschenett et al., 2003 | NPY2 | KO | Mice | EPM, OF, D/L, FST | KO displayed less anxiety-like behavior in EPM, OF, and light/dark box than WT. KO had less immobility than WT in FST. |  |
| Painsipp et al., 2008 | NPY2 | KO | Mice | OF, EPM, SIH, TST, object recognition | KO displayed less anxiety-like behavior than WT. KO had less immobility than WT in TST. |  |
|  | NPY4 | KO |  |  | KO displayed less anxiety-like behavior than WT. KO had less immobility than WT in TST. |  |
| Tasan et al., 2009 | NPY4 | KO | Mice | OF, EPM, D/L, SIH, FST, TST | Reduction of anxiety-like behaviors seen in light/dark box, but not EPM in KO compared to WT. KO had reduced immobility in both FST and TST. |  |
| Simen et al., 2006 | TNFR1 | KO | Mice | EPM, OF, D/L, FST, hot plate, CFC, SPT | KO had less immobility in FST than WT. No differences in anhedonia. No differences in anxiety-like behavior. |  |
|  | TNFR2 | KO |  |  | KO had less immobility in FST than WT. KO had less anhedonia than WT. No differences in anxiety-like behavior. |  |

5HT – serotonergic receptor; A – adenosine receptor; AC – adenylyl cyclase; Ar – aromatase; AR – adrenergic receptor; BDNF – brain-derived neurotrophic factor; CamKII – calcium/calmodulin-dependent protein kinase; CFC – contextual/cued fear conditioning; CREB – cyclic AMP response element binding protein; CRF – corticotropin-releasing factor; D – dopaminergic receptor; DAT – dopaminergic transporter; D/L – dark/light emergence test; ECT – electroconvulsive therapy; EOM – elevated zero maze; EPM – elevated plus maze; FST – forced swim test; GABA – γ-aminobutyric acid; GAT – GABAergic transporter; GalR – galanin receptor; GDNF – glial cell-derived neurotrophic factor; GR – glucocorticoid receptor; HDAC – histone deacetylase; Het – heterozygous; HPC – hippocampus; KO – knockout; LH – learned helplessness; MAO – monoamine oxidase; mGlu – metabotropic glutamate receptor; MT – melatonin receptor; NCAM – neural cell adhesion molecule; NET – noradrenergic transporter; NK – substance P; NOS – nitric oxide synthase; NPY – neuropeptide Y; NR – NMDA receptor; NSF – novelty suppressed feeding; OE – overexpressed; OF – open field; OR – opioid receptor; PACAP – pituitary adenylate cyclase-activating polypeptide; PPI – prepulse inhibition; R/I – resident intruder; SERT – serotonergic transporter; SIH – stressed-induced hyperthermia; SPT – sucrose preference test; SSRI – selective serotonin reuptake inhibitor; TCA – tricyclic antidepressant; TNFR – tumor necrosis factor α receptor; TPH – tryptophan hydroxylase 2; TST – tail suspension test; trkB – tropomyosin related kinase B; Ucn – urocortin

**Supplementary References**

Adachi, M., Barrot, M., Autry, A.E., Theobald, D., and Monteggia, L.M. (2008). Selective loss of brain-derived neurotrophic factor in the dentate gyrus attenuates antidepressant efficacy. *Biol Psychiatry* 63**,** 642-649.

Alexandre, C., Popa, D., Fabre, V., Bouali, S., Venault, P., Lesch, K.P., Hamon, M., and Adrien, J. (2006). Early life blockade of 5-hydroxytryptamine 1A receptors normalizes sleep and depression-like behavior in adult knock-out mice lacking the serotonin transporter. *J Neurosci* 26**,** 5554-5564.

Anttila, S., Viikki, M., Huuhka, K., Huuhka, M., Huhtala, H., Rontu, R., Lehtimaki, T., and Leinonen, E. (2009). TPH2 polymorphisms may modify clinical picture in treatment-resistant depression. *Neurosci Lett* 464**,** 43-46.

Aonurm-Helm, A., Jurgenson, M., Zharkovsky, T., Sonn, K., Berezin, V., Bock, E., and Zharkovsky, A. (2008). Depression-like behaviour in neural cell adhesion molecule (NCAM)-deficient mice and its reversal by an NCAM-derived peptide, FGL. *Eur J Neurosci* 28**,** 1618-1628.

Bale, T.L., and Vale, W.W. (2003). Increased depression-like behaviors in corticotropin-releasing factor receptor-2-deficient mice: sexually dichotomous responses. *J Neurosci* 23**,** 5295-5301.

Bhatnagar, S., Nowak, N., Babich, L., and Bok, L. (2004). Deletion of the 5-HT3 receptor differentially affects behavior of males and females in the Porsolt forced swim and defensive withdrawal tests. *Behav Brain Res* 153**,** 527-535.

Boulle, F., Van Den Hove, D.L., Jakob, S.B., Rutten, B.P., Hamon, M., Van Os, J., Lesch, K.P., Lanfumey, L., Steinbusch, H.W., and Kenis, G. (2012). Epigenetic regulation of the BDNF gene: implications for psychiatric disorders. *Mol Psychiatry* 17**,** 584-596.

Boyce-Rustay, J.M., and Holmes, A. (2006). Genetic inactivation of the NMDA receptor NR2A subunit has anxiolytic- and antidepressant-like effects in mice. *Neuropsychopharmacology* 31**,** 2405-2414.

Bukh, J.D., Bock, C., Vinberg, M., Werge, T., Gether, U., and Kessing, L.V. (2010). No interactions between genetic polymorphisms and stressful life events on outcome of antidepressant treatment. *Eur Neuropsychopharmacol* 20**,** 327-335.

Cases, O., Seif, I., Grimsby, J., Gaspar, P., Chen, K., Pournin, S., Muller, U., Aguet, M., Babinet, C., Shih, J.C., and Et Al. (1995). Aggressive behavior and altered amounts of brain serotonin and norepinephrine in mice lacking MAOA. *Science* 268**,** 1763-1766.

Chen, A., Zorrilla, E., Smith, S., Rousso, D., Levy, C., Vaughan, J., Donaldson, C., Roberts, A., Lee, K.F., and Vale, W. (2006). Urocortin 2-deficient mice exhibit gender-specific alterations in circadian hypothalamus-pituitary-adrenal axis and depressive-like behavior. *J Neurosci* 26**,** 5500-5510.

Chmielarz, P., Kusmierczyk, J., Parlato, R., Schutz, G., Nalepa, I., and Kreiner, G. (2013). Inactivation of glucocorticoid receptor in noradrenergic system influences anxiety- and depressive-like behavior in mice. *PLoS One* 8**,** e72632.

Cryan, J.F., Kelly, P.H., Neijt, H.C., Sansig, G., Flor, P.J., and Van Der Putten, H. (2003). Antidepressant and anxiolytic-like effects in mice lacking the group III metabotropic glutamate receptor mGluR7. *Eur J Neurosci* 17**,** 2409-2417.

Dalla, C., Antoniou, K., Papadopoulou-Daifoti, Z., Balthazart, J., and Bakker, J. (2004). Oestrogen-deficient female aromatase knockout (ArKO) mice exhibit depressive-like symptomatology. *Eur J Neurosci* 20**,** 217-228.

David, D.J., Samuels, B.A., Rainer, Q., Wang, J.W., Marsteller, D., Mendez, I., Drew, M., Craig, D.A., Guiard, B.P., Guilloux, J.P., Artymyshyn, R.P., Gardier, A.M., Gerald, C., Antonijevic, I.A., Leonardo, E.D., and Hen, R. (2009). Neurogenesis-dependent and -independent effects of fluoxetine in an animal model of anxiety/depression. *Neuron* 62**,** 479-493.

Doze, V.A., Handel, E.M., Jensen, K.A., Darsie, B., Luger, E.J., Haselton, J.R., Talbot, J.N., and Rorabaugh, B.R. (2009). alpha(1A)- and alpha(1B)-adrenergic receptors differentially modulate antidepressant-like behavior in the mouse. *Brain Res* 1285**,** 148-157.

Duncan, L.E., Hutchison, K.E., Carey, G., and Craighead, W.E. (2009). Variation in brain-derived neurotrophic factor (BDNF) gene is associated with symptoms of depression. *J Affect Disord* 115**,** 215-219.

Dziedzicka-Wasylewska, M., Faron-Gorecka, A., Kusmider, M., Drozdowska, E., Rogoz, Z., Siwanowicz, J., Caron, M.G., and Bonisch, H. (2006). Effect of antidepressant drugs in mice lacking the norepinephrine transporter. *Neuropsychopharmacology* 31**,** 2424-2432.

El Yacoubi, M., Ledent, C., Parmentier, M., Bertorelli, R., Ongini, E., Costentin, J., and Vaugeois, J.M. (2001). Adenosine A2A receptor antagonists are potential antidepressants: evidence based on pharmacology and A2A receptor knockout mice. *Br J Pharmacol* 134**,** 68-77.

Elliott, E., Ezra-Nevo, G., Regev, L., Neufeld-Cohen, A., and Chen, A. (2010). Resilience to social stress coincides with functional DNA methylation of the Crf gene in adult mice. *Nat Neurosci* 13**,** 1351-1353.

Fanous, S., Hammer, R.P., Jr., and Nikulina, E.M. (2010). Short- and long-term effects of intermittent social defeat stress on brain-derived neurotrophic factor expression in mesocorticolimbic brain regions. *Neuroscience* 167**,** 598-607.

Fedorova, I.M., Jacobson, M.A., Basile, A., and Jacobson, K.A. (2003). Behavioral characterization of mice lacking the A3 adenosine receptor: sensitivity to hypoxic neurodegeneration. *Cell Mol Neurobiol* 23**,** 431-447.

Fell, M.J., Witkin, J.M., Falcone, J.F., Katner, J.S., Perry, K.W., Hart, J., Rorick-Kehn, L., Overshiner, C.D., Rasmussen, K., Chaney, S.F., Benvenga, M.J., Li, X., Marlow, D.L., Thompson, L.K., Luecke, S.K., Wafford, K.A., Seidel, W.F., Edgar, D.M., Quets, A.T., Felder, C.C., Wang, X., Heinz, B.A., Nikolayev, A., Kuo, M.S., Mayhugh, D., Khilevich, A., Zhang, D., Ebert, P.J., Eckstein, J.A., Ackermann, B.L., Swanson, S.P., Catlow, J.T., Dean, R.A., Jackson, K., Tauscher-Wisniewski, S., Marek, G.J., Schkeryantz, J.M., and Svensson, K.A. (2011). N-(4-((2-(trifluoromethyl)-3-hydroxy-4-(isobutyryl)phenoxy)methyl)benzyl)-1-methy l-1H-imidazole-4-carboxamide (THIIC), a novel metabotropic glutamate 2 potentiator with potential anxiolytic/antidepressant properties: in vivo profiling suggests a link between behavioral and central nervous system neurochemical changes. *J Pharmacol Exp Ther* 336**,** 165-177.

Ferres-Coy, A., Santana, N., Castane, A., Cortes, R., Carmona, M.C., Toth, M., Montefeltro, A., Artigas, F., and Bortolozzi, A. (2013). Acute 5-HT(1)A autoreceptor knockdown increases antidepressant responses and serotonin release in stressful conditions. *Psychopharmacology (Berl)* 225**,** 61-74.

Filliol, D., Ghozland, S., Chluba, J., Martin, M., Matthes, H.W., Simonin, F., Befort, K., Gaveriaux-Ruff, C., Dierich, A., Lemeur, M., Valverde, O., Maldonado, R., and Kieffer, B.L. (2000). Mice deficient for delta- and mu-opioid receptors exhibit opposing alterations of emotional responses. *Nat Genet* 25**,** 195-200.

Gourley, S.L., Kiraly, D.D., Howell, J.L., Olausson, P., and Taylor, J.R. (2008). Acute hippocampal brain-derived neurotrophic factor restores motivational and forced swim performance after corticosterone. *Biol Psychiatry* 64**,** 884-890.

Grimsby, J., Toth, M., Chen, K., Kumazawa, T., Klaidman, L., Adams, J.D., Karoum, F., Gal, J., and Shih, J.C. (1997). Increased stress response and beta-phenylethylamine in MAOB-deficient mice. *Nat Genet* 17**,** 206-210.

Guscott, M., Bristow, L.J., Hadingham, K., Rosahl, T.W., Beer, M.S., Stanton, J.A., Bromidge, F., Owens, A.P., Huscroft, I., Myers, J., Rupniak, N.M., Patel, S., Whiting, P.J., Hutson, P.H., Fone, K.C., Biello, S.M., Kulagowski, J.J., and Mcallister, G. (2005). Genetic knockout and pharmacological blockade studies of the 5-HT7 receptor suggest therapeutic potential in depression. *Neuropharmacology* 48**,** 492-502.

Haller, J., Bakos, N., Rodriguiz, R.M., Caron, M.G., Wetsel, W.C., and Liposits, Z. (2002). Behavioral responses to social stress in noradrenaline transporter knockout mice: effects on social behavior and depression. *Brain Res Bull* 58**,** 279-284.

Hashimoto, H., Hashimoto, R., Shintani, N., Tanaka, K., Yamamoto, A., Hatanaka, M., Guo, X., Morita, Y., Tanida, M., Nagai, K., Takeda, M., and Baba, A. (2009). Depression-like behavior in the forced swimming test in PACAP-deficient mice: amelioration by the atypical antipsychotic risperidone. *J Neurochem* 110**,** 595-602.

Heisler, L.K., Chu, H.M., Brennan, T.J., Danao, J.A., Bajwa, P., Parsons, L.H., and Tecott, L.H. (1998). Elevated anxiety and antidepressant-like responses in serotonin 5-HT1A receptor mutant mice. *Proc Natl Acad Sci U S A* 95**,** 15049-15054.

Holmes, A., Hollon, T.R., Gleason, T.C., Liu, Z., Dreiling, J., Sibley, D.R., and Crawley, J.N. (2001). Behavioral characterization of dopamine D5 receptor null mutant mice. *Behav Neurosci* 115**,** 1129-1144.

Holmes, A., Yang, R.J., Murphy, D.L., and Crawley, J.N. (2002). Evaluation of antidepressant-related behavioral responses in mice lacking the serotonin transporter. *Neuropsychopharmacology* 27**,** 914-923.

Ibarguen-Vargas, Y., Surget, A., Touma, C., Palme, R., and Belzung, C. (2008). Multifaceted strain-specific effects in a mouse model of depression and of antidepressant reversal. *Psychoneuroendocrinology* 33**,** 1357-1368.

Ibarguen-Vargas, Y., Surget, A., Vourc'h, P., Leman, S., Andres, C.R., Gardier, A.M., and Belzung, C. (2009). Deficit in BDNF does not increase vulnerability to stress but dampens antidepressant-like effects in the unpredictable chronic mild stress. *Behav Brain Res* 202**,** 245-251.

Inta, D., Vogt, M.A., Luoni, A., Filipovic, D., Lima-Ojeda, J.M., Pfeiffer, N., Gasparini, F., Riva, M.A., and Gass, P. (2013). Significant increase in anxiety during aging in mGlu5 receptor knockout mice. *Behav Brain Res* 241**,** 27-31.

Jones, M.D., and Lucki, I. (2005). Sex differences in the regulation of serotonergic transmission and behavior in 5-HT receptor knockout mice. *Neuropsychopharmacology* 30**,** 1039-1047.

Keeney, A.J., Hogg, S., and Marsden, C.A. (2001). Alterations in core body temperature, locomotor activity, and corticosterone following acute and repeated social defeat of male NMRI mice. *Physiol Behav* 74**,** 177-184.

Krishnan, V., Graham, A., Mazei-Robison, M.S., Lagace, D.C., Kim, K.S., Birnbaum, S., Eisch, A.J., Han, P.L., Storm, D.R., Zachariou, V., and Nestler, E.J. (2008). Calcium-sensitive adenylyl cyclases in depression and anxiety: behavioral and biochemical consequences of isoform targeting. *Biol Psychiatry* 64**,** 336-343.

Lira, A., Zhou, M., Castanon, N., Ansorge, M.S., Gordon, J.A., Francis, J.H., Bradley-Moore, M., Lira, J., Underwood, M.D., Arango, V., Kung, H.F., Hofer, M.A., Hen, R., and Gingrich, J.A. (2003). Altered depression-related behaviors and functional changes in the dorsal raphe nucleus of serotonin transporter-deficient mice. *Biol Psychiatry* 54**,** 960-971.

Liu, G.X., Cai, G.Q., Cai, Y.Q., Sheng, Z.J., Jiang, J., Mei, Z., Wang, Z.G., Guo, L., and Fei, J. (2007). Reduced anxiety and depression-like behaviors in mice lacking GABA transporter subtype 1. *Neuropsychopharmacology* 32**,** 1531-1539.

Lu, X., Ross, B., Sanchez-Alavez, M., Zorrilla, E.P., and Bartfai, T. (2008). Phenotypic analysis of GalR2 knockout mice in anxiety- and depression-related behavioral tests. *Neuropeptides* 42**,** 387-397.

Macqueen, G.M., Ramakrishnan, K., Croll, S.D., Siuciak, J.A., Yu, G., Young, L.T., and Fahnestock, M. (2001). Performance of heterozygous brain-derived neurotrophic factor knockout mice on behavioral analogues of anxiety, nociception, and depression. *Behav Neurosci* 115**,** 1145-1153.

Mayorga, A.J., Dalvi, A., Page, M.E., Zimov-Levinson, S., Hen, R., and Lucki, I. (2001). Antidepressant-like behavioral effects in 5-hydroxytryptamine(1A) and 5-hydroxytryptamine(1B) receptor mutant mice. *J Pharmacol Exp Ther* 298**,** 1101-1107.

Miyamoto, Y., Yamada, K., Noda, Y., Mori, H., Mishina, M., and Nabeshima, T. (2002). Lower sensitivity to stress and altered monoaminergic neuronal function in mice lacking the NMDA receptor epsilon 4 subunit. *J Neurosci* 22**,** 2335-2342.

Mombereau, C., Kaupmann, K., Froestl, W., Sansig, G., Van Der Putten, H., and Cryan, J.F. (2004). Genetic and pharmacological evidence of a role for GABA(B) receptors in the modulation of anxiety- and antidepressant-like behavior. *Neuropsychopharmacology* 29**,** 1050-1062.

Mombereau, C., Kaupmann, K., Gassmann, M., Bettler, B., Van Der Putten, H., and Cryan, J.F. (2005). Altered anxiety and depression-related behaviour in mice lacking GABAB(2) receptor subunits. *Neuroreport* 16**,** 307-310.

Monteggia, L.M., Luikart, B., Barrot, M., Theobold, D., Malkovska, I., Nef, S., Parada, L.F., and Nestler, E.J. (2007). Brain-derived neurotrophic factor conditional knockouts show gender differences in depression-related behaviors. *Biol Psychiatry* 61**,** 187-197.

Murray, F., Smith, D.W., and Hutson, P.H. (2008). Chronic low dose corticosterone exposure decreased hippocampal cell proliferation, volume and induced anxiety and depression like behaviours in mice. *Eur J Pharmacol* 583**,** 115-127.

Newton, S.S., Thome, J., Wallace, T.L., Shirayama, Y., Schlesinger, L., Sakai, N., Chen, J., Neve, R., Nestler, E.J., and Duman, R.S. (2002). Inhibition of cAMP response element-binding protein or dynorphin in the nucleus accumbens produces an antidepressant-like effect. *J Neurosci* 22**,** 10883-10890.

Painsipp, E., Wultsch, T., Edelsbrunner, M.E., Tasan, R.O., Singewald, N., Herzog, H., and Holzer, P. (2008). Reduced anxiety-like and depression-related behavior in neuropeptide Y Y4 receptor knockout mice. *Genes Brain Behav* 7**,** 532-542.

Ramboz, S., Oosting, R., Amara, D.A., Kung, H.F., Blier, P., Mendelsohn, M., Mann, J.J., Brunner, D., and Hen, R. (1998). Serotonin receptor 1A knockout: an animal model of anxiety-related disorder. *Proc Natl Acad Sci U S A* 95**,** 14476-14481.

Razzoli, M., Carboni, L., Andreoli, M., Michielin, F., Ballottari, A., and Arban, R. (2011). Strain-specific outcomes of repeated social defeat and chronic fluoxetine treatment in the mouse. *Pharmacol Biochem Behav* 97**,** 566-576.

Richardson-Jones, J.W., Craige, C.P., Guiard, B.P., Stephen, A., Metzger, K.L., Kung, H.F., Gardier, A.M., Dranovsky, A., David, D.J., Beck, S.G., Hen, R., and Leonardo, E.D. (2010). 5-HT1A autoreceptor levels determine vulnerability to stress and response to antidepressants. *Neuron* 65**,** 40-52.

Ridder, S., Chourbaji, S., Hellweg, R., Urani, A., Zacher, C., Schmid, W., Zink, M., Hortnagl, H., Flor, H., Henn, F.A., Schutz, G., and Gass, P. (2005). Mice with genetically altered glucocorticoid receptor expression show altered sensitivity for stress-induced depressive reactions. *J Neurosci* 25**,** 6243-6250.

Rupniak, N.M., Carlson, E.J., Webb, J.K., Harrison, T., Porsolt, R.D., Roux, S., De Felipe, C., Hunt, S.P., Oates, B., and Wheeldon, A. (2001). Comparison of the phenotype of NK1R-/- mice with pharmacological blockade of the substance P (NK1 ) receptor in assays for antidepressant and anxiolytic drugs. *Behav Pharmacol* 12**,** 497-508.

Sallinen, J., Haapalinna, A., Macdonald, E., Viitamaa, T., Lahdesmaki, J., Rybnikova, E., Pelto-Huikko, M., Kobilka, B.K., and Scheinin, M. (1999). Genetic alteration of the alpha2-adrenoceptor subtype c in mice affects the development of behavioral despair and stress-induced increases in plasma corticosterone levels. *Mol Psychiatry* 4**,** 443-452.

Sarkisyan, G., Roberts, A.J., and Hedlund, P.B. (2010). The 5-HT(7) receptor as a mediator and modulator of antidepressant-like behavior. *Behav Brain Res* 209**,** 99-108.

Schramm, N.L., Mcdonald, M.P., and Limbird, L.E. (2001). The alpha(2a)-adrenergic receptor plays a protective role in mouse behavioral models of depression and anxiety. *J Neurosci* 21**,** 4875-4882.

Schweizer, M.C., Henniger, M.S., and Sillaber, I. (2009). Chronic mild stress (CMS) in mice: of anhedonia, 'anomalous anxiolysis' and activity. *PLoS One* 4**,** e4326.

Simen, B.B., Duman, C.H., Simen, A.A., and Duman, R.S. (2006). TNFalpha signaling in depression and anxiety: behavioral consequences of individual receptor targeting. *Biol Psychiatry* 59**,** 775-785.

Smith, C.M., Lawrence, A.J., Sutton, S.W., and Gundlach, A.L. (2009). Behavioral phenotyping of mixed background (129S5:B6) relaxin-3 knockout mice. *Ann N Y Acad Sci* 1160**,** 236-241.

Solomon, M.B., Furay, A.R., Jones, K., Packard, A.E., Packard, B.A., Wulsin, A.C., and Herman, J.P. (2012). Deletion of forebrain glucocorticoid receptors impairs neuroendocrine stress responses and induces depression-like behavior in males but not females. *Neuroscience* 203**,** 135-143.

Spielewoy, C., Roubert, C., Hamon, M., Nosten-Bertrand, M., Betancur, C., and Giros, B. (2000). Behavioural disturbances associated with hyperdopaminergia in dopamine-transporter knockout mice. *Behav Pharmacol* 11**,** 279-290.

Tanda, K., Nishi, A., Matsuo, N., Nakanishi, K., Yamasaki, N., Sugimoto, T., Toyama, K., Takao, K., and Miyakawa, T. (2009). Abnormal social behavior, hyperactivity, impaired remote spatial memory, and increased D1-mediated dopaminergic signaling in neuronal nitric oxide synthase knockout mice. *Mol Brain* 2**,** 19.

Tasan, R.O., Lin, S., Hetzenauer, A., Singewald, N., Herzog, H., and Sperk, G. (2009). Increased novelty-induced motor activity and reduced depression-like behavior in neuropeptide Y (NPY)-Y4 receptor knockout mice. *Neuroscience* 158**,** 1717-1730.

Taylor, M.J., Sen, S., and Bhagwagar, Z. (2010). Antidepressant response and the serotonin transporter gene-linked polymorphic region. *Biol Psychiatry* 68**,** 536-543.

Tordera, R.M., Garcia-Garcia, A.L., Elizalde, N., Segura, V., Aso, E., Venzala, E., Ramirez, M.J., and Del Rio, J. (2011). Chronic stress and impaired glutamate function elicit a depressive-like phenotype and common changes in gene expression in the mouse frontal cortex. *Eur Neuropsychopharmacol* 21**,** 23-32.

Tronche, F., Kellendonk, C., Kretz, O., Gass, P., Anlag, K., Orban, P.C., Bock, R., Klein, R., and Schutz, G. (1999). Disruption of the glucocorticoid receptor gene in the nervous system results in reduced anxiety. *Nat Genet* 23**,** 99-103.

Tschenett, A., Singewald, N., Carli, M., Balducci, C., Salchner, P., Vezzani, A., Herzog, H., and Sperk, G. (2003). Reduced anxiety and improved stress coping ability in mice lacking NPY-Y2 receptors. *Eur J Neurosci* 18**,** 143-148.

Uchida, S., Hara, K., Kobayashi, A., Otsuki, K., Yamagata, H., Hobara, T., Suzuki, T., Miyata, N., and Watanabe, Y. (2011). Epigenetic status of Gdnf in the ventral striatum determines susceptibility and adaptation to daily stressful events. *Neuron* 69**,** 359-372.

Van Gaalen, M.M., Stenzel-Poore, M.P., Holsboer, F., and Steckler, T. (2002). Effects of transgenic overproduction of CRH on anxiety-like behaviour. *Eur J Neurosci* 15**,** 2007-2015.

Weil, Z.M., Hotchkiss, A.K., Gatien, M.L., Pieke-Dahl, S., and Nelson, R.J. (2006). Melatonin receptor (MT1) knockout mice display depression-like behaviors and deficits in sensorimotor gating. *Brain Res Bull* 68**,** 425-429.

Weisstaub, N.V., Zhou, M., Lira, A., Lambe, E., Gonzalez-Maeso, J., Hornung, J.P., Sibille, E., Underwood, M., Itohara, S., Dauer, W.T., Ansorge, M.S., Morelli, E., Mann, J.J., Toth, M., Aghajanian, G., Sealfon, S.C., Hen, R., and Gingrich, J.A. (2006). Cortical 5-HT2A receptor signaling modulates anxiety-like behaviors in mice. *Science* 313**,** 536-540.

Xing, B., Liu, P., Jiang, W.H., Liu, F., Zhang, H., Cao, G.F., Chen, T., and Dang, Y.H. (2013). Effects of immobilization stress on emotional behaviors in dopamine D3 receptor knockout mice. *Behav Brain Res* 243**,** 261-266.

Yalcin, I., Belzung, C., and Surget, A. (2008). Mouse strain differences in the unpredictable chronic mild stress: a four-antidepressant survey. *Behav Brain Res* 193**,** 140-143.

Zeng, H., Schimpf, B.A., Rohde, A.D., Pavlova, M.N., Gragerov, A., and Bergmann, J.E. (2007). Thyrotropin-releasing hormone receptor 1-deficient mice display increased depression and anxiety-like behavior. *Mol Endocrinol* 21**,** 2795-2804.

Zhao, Y., Ma, R., Shen, J., Su, H., Xing, D., and Du, L. (2008). A mouse model of depression induced by repeated corticosterone injections. *Eur J Pharmacol* 581**,** 113-120.

Zorner, B., Wolfer, D.P., Brandis, D., Kretz, O., Zacher, C., Madani, R., Grunwald, I., Lipp, H.P., Klein, R., Henn, F.A., and Gass, P. (2003). Forebrain-specific trkB-receptor knockout mice: behaviorally more hyperactive than "depressive". *Biol Psychiatry* 54**,** 972-982.
